# Factors associated with reported service use for mental health problems by residents of rural and remote communities: cross-sectional findings from a baseline survey

**DOI:** 10.1186/1472-6963-13-157

**Published:** 2013-04-30

**Authors:** David Perkins, Jeffrey Fuller, Brian J Kelly, Terry J Lewin, Michael Fitzgerald, Clare Coleman, Kerry J Inder, John Allan, Dinesh Arya, Russell Roberts, Richard Buss

**Affiliations:** 1 Centre of Research Excellence in Rural and Remote Primary Health Care and Department of Rural Health, University of Sydney, Broken Hill, NSW, Australia; 2 School of Nursing and Midwifery, Flinders University, Adelaide, SA, Australia; 3 Centre for Translational Neuroscience and Mental Health, University of Newcastle, Newcastle, NSW, Australia; 4 Mental Health Services, Hunter New England Local Health District, Newcastle, NSW, Australia; 5 Centre for Epidemiology and Biostatistics, University of Newcastle, Newcastle, NSW, Australia; 6 Hunter Medical Research Institute, Newcastle, NSW, Australia; 7 Centre for Rural and Remote Mental Health, University of Newcastle, Orange, NSW, Australia; 8 Mental Health and Drug & Alcohol Office, NSW Health, Sydney, Australia; 9 Department of Health, Northern Territory Government, Darwin, Australia; 10 Mental Health and Drug and Alcohol, Western NSW Local Health District, Dubbo, NSW, Australia; 11 Mental Health Services, Northern NSW Local Health District, Lismore, NSW, Australia

**Keywords:** Health service utilisation, Mental health, Rural health

## Abstract

**Background:**

The patterns of health service use by rural and remote residents are poorly understood and under-represented in national surveys. This paper examines professional and non-professional service use for mental health problems in rural and remote communities in Australia.

**Methods:**

A stratified random sample of adults was drawn from non-metropolitan regions of New South Wales, Australia as part of a longitudinal population-based cohort. One-quarter (27.7%) of the respondents were from remote or very remote regions. The socio-demographic, health status and service utilization (professional and non-professional) characteristics of 2150 community dwelling residents are described. Hierarchical logistic regressions were used to identify cross-sectional associations between socio-demographic, health status and professional and non-professional health service utilization variables.

**Results:**

The overall rate of professional contacts for mental health problems during the previous 12 months (17%) in this rural population exceeded the national rate (11.9%). Rates for psychologists and psychiatrists were similar but rates for GPs were higher (12% vs. 8.1%). Non-professional contact rates were 12%. Higher levels of help seeking were associated with the absence of a partner, poorer finances, severity of mental health problems, and higher levels of adversity. Remoteness was associated with lower utilization of non-professional support. A Provisional Service Need Index was devised, and it demonstrated a broad dose–response relationship between severity of mental health problems and the likelihood of seeking any professional or non-professional help. Nevertheless, 47% of those with estimated high service need had no contact with professional services.

**Conclusions:**

An examination of self-reported patterns of professional and non-professional service use for mental health problems in a rural community cohort revealed relatively higher rates of general practitioner attendance for such problems compared with data from metropolitan centres. Using a measure of Provisional Service Need those with greater needs were more likely to access specialist services, even in remote regions, although a substantial proportion of those with the highest service need sought no professional help. Geographic and financial barriers to service use were identified and perception of service adequacy was relatively low, especially among those with the highest levels of distress and greatest adversity.

## Background

There is a growing body of research about the use of formal and informal support services for mental health problems. For example, recent national surveys from Australia [1], USA [2], England [3] and Canada [4] have documented prevalence rates for mental disorders, professional and non-professional service usage patterns [5-9], perceived need profiles [10-12], and associated linkages to sources of mental health information [13]. However, evidence about the prevalence of mental disorders, symptoms, and patterns of service use in rural and remote communities is in short supply for a variety of reasons. Surveying residents of these regions is expensive and difficult. Indeed, the 2007 Australian National Survey of Mental Health and Wellbeing (NSMHWB) acknowledged that it was ‘… limited in its representation of people from remote and very remote areas’ ([1], p. 597) while in some Canadian provinces oversampling has been used in the national survey to provide reliable sub-provincial prevalence estimates [4].

The meaning of rurality varies and there are difficulties in assessing evidence from countries with substantial rural populations such as Scotland, Australia, Canada or the United States. Some authors [14] have concluded that the definition and official classifications of rurality are not helpful in making meaningful distinctions between communities and that we should either change the classifications [15] or stop using them; for example Rost et al. proposed ‘… that investigators no longer employ any of the multiple definitions of rurality proposed in the literature [14] (p. 232).

There are a number of reports that conclude that the prevalence of mental health symptoms is similar between rural and urban areas [16-18]. Likewise, Smith [19] reports that ‘there is little evidence linking prevalence of mental health disorders with rurality’ (p. 58). Some authors, such as Ziller [20], found that self reported need for mental health services was slightly (but statistically significantly) higher in rural US. However, there are a number of reasons to believe that the use of services for mental health problems may differ between rural/remote residents and their urban peers including: specialist workforce shortages in rural areas [21]; generalist workforce shortages in remote and very remote areas [21,22]; costs, travel and waiting times for rural and remote patients [23]; culture and attitudinal factors including “rural stoicism”, concerns about privacy and confidentiality in small communities [24]; and, lower rates of engagement in and continuation with treatment for mental illness in rural areas [14].

In a study of 78,000 employees in Australia, Hilton et al. [25] found, perhaps unsurprisingly, that those in very remote regions were less likely to access mental health treatments. Parslow et al. [7] compared service use in Australia using the 1997 and 2007 NSMHWB and concluded that use of any mental health service had risen significantly but that the proportion seeing General Practitioners (GPs) for mental health services had not increased (p. 206). This increase in mental health service use has been influenced by Federal programs to improve access to mental health practitioners, including the Access to Allied Psychological Services and the Better Access to Psychiatrists, Psychologists and General Practitioners initiatives introduced in 2001 and 2006 respectively [26]. GPs are key providers in rural areas with specialist shortages and they are the first choice for many people seeking help for a mental health problem [26].

While Mechanic [27] argues that we cannot use prevalence of mental disorders as a proxy for service need, the aggregate pattern of service use in Australia and elsewhere by those with mental health disorders would appear to be one of underuse [28]. The 2007 Australian NSMHWB concluded that ‘rates of service use for people with a mental health condition are less than optimal [5] (p. 615). About one-third (34.9%) of those meeting the Composite International Diagnostic Interview (CIDI) criteria for a mental disorder reported that they received services in the previous 12 months. There was evidence that those with severe disorders made more use of community based mental health services (63.5%) than those with mild disorders (17.7%). The extent of this “underuse” is not clear, since no-one is suggesting that all those with a mental health disorder identified in a research interview would benefit from treatment from a generalist or a mental health specialist. In another Australian study, Short et al. [29] concluded that public mental health services care for people with psychotic disorders while ‘those with high prevalence disorders … seek psychiatric treatment elsewhere’ (p. 475).

It is important to acknowledge that the Australian health care system differs from unitary health care systems, such as those in the UK and New Zealand, having a state-federal separation of specialist and primary mental health care funding and responsibilities. Unitary systems are better able to integrate specialist and primary care based services since they have population based planning and funding arrangements. In Australia, access to services can be compromised since there is no effective process for joint or shared planning [30].

There is some international evidence about non-specialist sources of care such as religious advisors [31] or self care [32]. Verhaak et al. [33] note that patients may not recognise that their problems have a mental health origin or may question the effectiveness of their providers. In a cross-European survey of adults from six nations, ten Have et al. [34] reported a low perceived effectiveness of professional care and about a third of respondents believed it was worse than no care for those with serious emotional problems. In the Australian context, Olesen et al. [6] concluded that there is widespread use of non-professional services and self-management strategies (e.g., 33.5% of adults with an affective or anxiety disorder in the last 12 months sought help from family or friends). These findings are consistent with those from studies investigating community attitudes to mental illness in general, with low rates of confidence in professional treatment, as a component of the prevailing stigma of mental disorders [35].

Some American studies have examined whether rural residents have access to an appropriate range and “dose” of services. Fortney reported that about 60% of a rural sample with self-reported depression received some formal treatment, with more than 50% receiving pharmacotherapy and 25% receiving psychotherapy [36,37]. The rates of minimally adequate treatment were measured as the number of scripts filled and the number of psychotherapy sessions attended [36]. He noted that if these rural residents do not respond to medication they may not have access to psychotherapy as an alternative treatment. In a study of American veterans with depression, anxiety or post traumatic stress disorder, Cully et al. [38] found that rural veterans were less likely to receive psychotherapy than their urban peers and that the “dose” was less likely to be adequate. While shortages of specialist mental health staff characterise rural and remote communities in Australia, it is not clear if these findings hold true.

In a study of depression and remoteness in South Australia, Goldney [17] found that rates of treatment of depression with antidepressants were similar in less accessible and more accessible areas and that a higher proportion of the rural depressed had contact with a health service, social worker or counsellor than their more accessible peers. Diagnostic interviews were not used in this study; hence, a limitation was the reliance on self-identification of past “depression”. Jackson et al. [39] have identified factors contributing to mental health usage in a rural setting, including gender, age, marital status, mental disorder, co-morbidity and psychological distress. Similarly, in the 2007 Australian NSMHWB, service usage among adults with a mental health problem was lowest among males and the youngest and oldest age groups [5,6].

In summary, the use of services for mental health disorders presents a complex picture. A focus on rural and remote settings complicates matters and international evidence is hard to compare. Given that access to services for mental health problems is limited in rural and remote regions, questions remain about from whom rural residents in Australia seek help, how frequently this occurs and what characteristics are associated with this help-seeking behaviour. This paper examines the impact of remoteness on the patterns of service use for mental health problems in a rural community sample, how they compare with national data for urban residents from the 2007 Australian NSMHWB, and the relationship between self-reported service use and estimates of the potential need for services.

### Heath service context

Australia is a federation of 6 states and 2 territories, where public hospitals are funded by state departments of health and admission to public hospitals is free. The majority of GPs and medical specialists, including psychiatrists, are self-employed and their fees are subsidised or paid in full by the Federal Medical Insurance system. GPs act as gatekeepers for accessing specialist services, and patients can be referred to psychiatrists, psychologists and other allied health professionals, including social workers, mental health nurses and occupational therapists [26].

While the number of full-time equivalent GPs per 100,000 members of the population is reasonably similar across all categories of remoteness in Australia, the number of medical specialists decreases with increasing remoteness. For example, there are approximately 18 full-time equivalent psychiatrists per 100,000 people comprising 23 (per 100,000) in major cities, compared to 7 for inner regional, 5 for outer regional, and 3 for remote and very remote areas [40]. In rural and remote areas, access to GPs is also complicated by distance and sometimes by cost, through the use of co-payments.

## Methods

Complete details of the methodology and measures used in the Australian Rural Mental Health Study (ARMHS), a longitudinal population-based cohort, are provided elsewhere [41,42]. This project was approved by the Human Research Ethics Committees of the University of Newcastle, University of Sydney, Greater Western Area Health Service, Hunter New England Area Health Service and the North Coast Area Health Service.

### Recruitment

Sixty Local Government Areas were identified from three Australian rural health service regions of the state of New South Wales (NSW) using the Rural, Remote and Metropolitan Areas classification, representing approximately 70% of the geographic region of non-metropolitan NSW. Over-sampling of the remote and very remote regions ensured sufficient sample size from these regions. The baseline sample was recruited between 2007 and 2009 and comprised residents aged 18 years or older living in private dwellings. Households were identified from Australian electoral rolls and sent a letter informing them of the study. Matching telephone numbers were found from an electronic directory and households were called requesting consent for the study and to identify a contact person. Consent papers and questionnaires were sent to those who agreed to participate.

Participants aged 65 years or over were screened for cognitive impairment using the modified Telephone Interview for Cognitive Status (TICS-M) [43] and those with a TICS-M total score < 17 were excluded. Non-English speaking members of a household, those with significant hearing impairment that impeded consent and/or interview, and those with no identifiable telephone contact number (after directory and electronic database search) were also excluded. The baseline survey used self report measures, administered by post in two parts (survey A and B) mailed two weeks apart and excluded special dwellings (such as hospitals, nursing homes, prisons, hotels and hostels) and overseas visitors usually resident outside Australia.

### Variables and instruments

#### Socio-demographic characteristics

Basic socio-demographic questions were included in Survey A and the following characteristics were coded for the current analyses: age in years (grouped into five categories 18–34, 35–44, 45–54, 55–64 and 65+); gender (male, female); education (partial schooling, completed high school or above, and unknown); and marital status (married or de facto, divorced or separated, widowed, and never married). Financial position was assessed using the perceived prosperity item from the Household, Income and Labour Dynamics in Australia (HILDA) study (“Given your current needs and financial responsibilities, would you say that you and your family are: prosperous, very comfortable, reasonably comfortable, just getting along, poor, or very poor?”) [44]. Rurality/remoteness was categorised using the Australian Standard Geographic Classification (ASGC) system, which allocates classes of remoteness to localities, based on the Accessibility/Remoteness Index of Australia Plus (ARIA+) values: major cities, inner regional, outer regional, remote and very remote. The current study selected people residing in non-metropolitan areas, with the remaining categories used for classification of district level remoteness. ARIA+, developed by the National Centre for Social Applications of Geographic Information Systems (http://www.gisca.adelaide.edu.au), describes remoteness from goods and services for any part of Australia relying on road distance as a surrogate for remoteness and on the population size of a service centre as a surrogate for the availability of services [45].

#### Health status characteristics

For the current analyses, seven health characteristics were selected, which comprised direct global ratings about health, standardised measures frequently used in mental health epidemiological research, risk behaviours or circumstances linked with mental health (e.g., substance use, smoking, recent adversity). Specifically: single item self report measures were devised (for Survey A) to assess overall physical and overall mental health, using 5-point Likert scales ranging from poor to excellent; the Kessler K-10 (K-10) was used (in Survey A) to assess psychological distress [18,46]; the World Health Organisation’s ten item Alcohol Use Disorders Identification Test (AUDIT) was used (in Survey B) to measure current hazardous alcohol use, dichotomised as low risk (scores of 0–7) or at-risk (scores ≥ 8) [47,48]; the nine item Patient Health Questionnaire (PHQ-9) was used (in Survey B) to assess current depressive symptoms (at least one symptom for more than half the time) [49]; and a 12 item adverse life events scale was used (in Survey A) for events within the preceding 12 months [50], which was categorised as: 0–2, 3–5, or more than 5 events.

#### Service utilisation: self-reported professional and non-professional contacts for mental health

Recent service use for mental health problems was investigated primarily using items from the 2007 Australian NSMHWB [1,5,10], which included: questions about the frequency of visits to a range of mental health professionals “… in the past 12 months regarding your own mental health problems, such as stress, anxiety or depression, or worries about alcohol or drugs”; and comparable questions about other contacts (e.g., with other professionals, friends or family, clergy, etc.). Questions about the forms of help received, their overall adequacy, and potential barriers were asked, including a measure of inadequate help – “In general, did you get as much help as you needed (yes or no)?” and factors that might have stopped or delayed access to health care when it was needed: distance to travel; cost; transport; limited choices; time taken to get an appointment; confidentiality; time away from work, carer responsibilities, stress and other. Also included in Survey B were a range of questions about other ways of accessing support for mental health problems during the past 12 months, including the frequency of using internet support groups, self-help groups, and telephone counselling.

### Data analysis

Data entry, cleaning, aggregation and analysis techniques primarily involved the Statistical Package for Social Sciences (SPSS version 17.0; Chicago, IL, USA) and SAS (SAS V9.2; SAS Institute Inc., Cary, NC, USA) statistical software. The initial analyses reported below are descriptive in nature, detailing the socio-demographic and health status characteristics of the participants who completed the service utilisation questions, followed by patterns of self-reported professional and non-professional contacts during the past 12 months (including the percentages with any contacts, and the number and distribution of contacts by service users). For the major analyses, hierarchical logistic regressions were used for the outcome variables of professional contact and non-professional contact, each coded as no contact (0) versus any contact (1). All predictor variables were categorical in nature, with socio-demographic characteristics entered simultaneously at step 1, followed by each of the individual health status characteristics entered separately at step 2 (as they tended to be interdependent) thereby assessing relationships between the various health status characteristics and service use, while controlling for any underlying socio-demographic influences. For the sub-group of participants who reported having contact with a professional or non-professional during the past 12 months for their own mental health problem, factors associated with inadequate help, defined as answering no to the question about receiving as much help as needed, were explored using logistic regression techniques. Participants were only included in these analyses if they had the full set of socio-demographic characteristics; and, as a partial control for the number of statistical tests, the threshold for significance was set at p < 0.01. These associations are reported as Odds Ratios (OR) with 99% Confidence Intervals (CI).

## Results

### Sample characteristics

The total ARMHS cohort comprised 2639 individuals (representing 1879 households), with marginally higher participation rates in remote regions (31% versus 27% overall); full details of the study sample are described by Kelly et al. [42]. Of the 2150 participants who completed the service utilisation questions in survey B (see Table 1), a higher proportion were female and aged over 65 years than the New South Wales rural population overall (30% compared with 23% for NSW). ARMHS also over sampled from remote and very remote regions to ensure that issues pertaining to these sub-populations were adequately represented. These sample characteristics need to be taken into account when considering the results.

**Table 1:** Participant characteristics of the baseline ARMHS sample responding to the service utilisation questions (N = 2150)

**Socio-demographic characteristic**	**n (%)**	**Current health status**	**n (%)**
Age (years):		Overall mental health:	
18-34	157 (7.3)	Good to excellent	1851 (87)
35-44	277 (13)	Poor/fair	279 (13)
45-54	452 (21)	Overall physical health:	
55-64	617 (29)	Good to excellent	1690 (79)
65+	647 (30)	Poor/fair	439 (21)
Gender:			
Male	853 (40)	Kessler K-10 score:	
Female	1297 (60)	10-15	1503 (71)
Education:		16-24	489 (23)
Partial schooling	605 (28)	25-50	132 (6.2)
Completed high school or above	1399 (65)		
Unknown	146 (6.8)	AUDIT:	
Marital status (n=2133):		0-7	1829 (86)
Married/de facto	1590 (75)	8+	297 (14)
Divorced/separated	215 (10)		
Widowed	172 (8.1)	PHQ-9 symptomatology:	
Never married	156 (7.3)	No symptoms	1718 (81)
ASGC category (Remoteness):		Depressive symptoms	415 (19)
Inner regional	778 (36)		
Outer regional	802 (37)	Recent adverse life events:	
Remote	406 (19)	0-2	1668 (80)
Very remote	164 (7.6)	3-5	363 (17)
Financial position (n=2126):		>5	48 (2.3)
Prosperous/comfortable	340 (16)		
Reasonable	1080 (51)	Smoking status:	
Just getting along to very poor	706 (33)	No	1858 (87)
		Yes	274 (13)

ARMHS, Australian Rural Mental Health Study; ASGC, Australian Standard Geographic Classification category, based on the Accessibility Remote Index of Australia (ARIA+ version); AUDIT, Alcohol Use Disorders Identification Test; PHQ-9, Patient Health questionnaire.

Table 1 describes measures of current health. The proportion of participants (29.2%) reporting moderate levels of current distress on the K-10 (score 16+) was comparable to the 2007 National Survey of Mental Health and Wellbeing [51]. Just under one-fifth (19%) showed current depressive symptoms on the PHQ-9 and had experienced more than three recent adverse life events (19.3%) in the past 12 months. These levels of morbidity and adversity are also consistent with the help seeking profiles detailed below, with just over a fifth (n = 445, 20.7%) reporting seeking some help for a mental health problem during the past 12 months.

### Contacts for mental health problems

More people reported professional than non-professional contacts (17.3% vs. 11.6%) in the past 12 months, with comparable mean annual contact rates by service users (professional contacts: 11.0; non-professional contacts: 11.3) (see Table 2). A total of 8.2% (n = 177) reported both professional and non-professional contacts in the past 12 months. A general practitioner (GP) was by far the main professional from whom help was sought, although the frequency of mental health related GP contacts (by service users) was lower than for a psychiatrist, psychologist or mental health nurse, which probably reflects the different levels of severity among people being treated. Surprisingly, very few participants reported seeing a drug and alcohol counsellor. Family and friends were the main source of non-professional help, accounting for almost 60% of such contacts (1664 of 2825 non-professional contacts).

**Table 2:** **Self-reported *professional and non-professional * contacts during the past 12 months for mental health problems (N = 2150)**

**Contact type**	**Any contacts n (%)**	**Number of times contacted (by service users)**	
		**Mean (SD)**	**Median (range)**
Professional contacts:			
Total	372 (17)	11.0 (33.5)	4.0 (1.0, 600.0)
General Practitioner (GP)	264 (12)	4.9 (7.9)	3.0 (1.0, 84.0)
Psychiatrist	54 (2.5)	6.7 (13.1)	3.5 (1.0, 96.0)
Psychologist	81 (3.8)	6.6 (6.3)	5.0 (1.0, 40.0)
Drug and alcohol counsellor	5 (0.2)	2.6 (2.1)	2.0 (1.0, 6.0)
Mental health nurse	24 (1.1)	8.5 (20.3)	2.5 (1.0, 96.0)
Other mental health professional	63 (2.9)	5.7 (7.3)	3.0 (1.0, 48.0)
Chemist	75 (3.5)	4.8 (11.5)	2.0 (1.0, 96.0)
Specialist doctor or surgeon	93 (4.3)	3.5 (9.1)	1.0 (1.0, 84.0)
Other professional providing general services	89 (4.1)	5.9 (7.8)	4.0 (1.0, 56.0)
Hospital admission (nights)	10 (0.5)	11.9 (9.5)	10.5 (2.0, 28.0)
Non-professional contacts:			
Total	250 (12)	11.3 (18.4)	4.5 (1.0, 125.0)
Alternative practitioner	39 (1.8)	3.6 (3.4)	2.0 (1.0, 18.0)
Friend or family	157 (7.3)	10.6 (15.5)	5.0 (1.0, 99.0)
Clergy	35 (1.6)	4.2 (4.6)	2.0 (1.0, 20.0)
Other persons	8 (0.4)	8.3 (8.7)	6.5 (1.0, 28.0)
			
All contacts (professional and non-professional)	445 (21)	15.6 (40.3)	5.0 (1.0, 725.0)

SD: Standard deviation.

### Service user profiles

Relationships between socio-demographic characteristics and reported contacts for mental health problems during the past 12 months (Table 3) show that those who had never married (OR = 1.98) or who were divorced or separated (OR = 2.12) had approximately double the odds of having had at least one professional contact for a mental health problem than those who were currently married. Not providing information about school completion was associated with higher odds of reporting professional contacts (OR = 2.01). Likewise, those who were “just getting along to very poor” financially were also more likely (OR = 2.23) to have sought help than those who were “prosperous/comfortable”. No differences were found on professional help seeking according to age, gender or rurality/remoteness.

**Table 3:** **Relationships between socio-demographic characteristics and reported *professional *and *non-professional *contacts for mental health problems (N = 2101)**

**Socio-demographic characteristic**	**Sub-group n**	**Professional service utilisation**		**Non-professional service utilisation**	
		**n (%)**	**OR (99% CI)**	**n (%)**	**OR (99% CI)**
Age (years):					
18-34	150	25 (17)		21 (14)	
35-44	275	72 (26)	1.93 (0.95, 3.93)	56 (20)	1.73 (0.81, 3.73)
45-54	445	87 (20)	1.32 (0.66, 2.64)	68 (15)	1.21 (0.57, 2.57)
55-64	597	100 (17)	1.19 (0.59, 2.37)	66 (11)	0.92 (0.43, 1.98)
65+	634	79 (12)	0.83 (0.40, 1.72)	30 (4.7)	0.34 (0.14, 0.83)*
Gender:					
Male	838	138 (16)		68 (8.1)	
Female	1263	225 (18)	1.11 (0.80, 1.53)	173 (14)	1.79 (1.19, 2.69)**
Education:					
Partial schooling	595	82 (14)		47 (7.9)	
Completed high school or above	1388	253 (18)	1.35 (0.92, 1.98)	180 (13)	1.47 (0.91, 2.36)
Unknown	118	28 (24)	2.01 (1.04, 3.87)*	14 (12)	1.74 (0.73, 4.15)
Marital status:					
Married/de facto	1570	233 (15)		152 (9.7)	
Divorced/separated	212	64 (30)	2.12 (1.36, 3.31)**	46 (22)	2.29 (1.38, 3.83)**
Widowed	168	25 (15)	1.18 (0.62, 2.26)	15 (8.9)	1.64 (0.72, 3.77)
Never married	151	41 (27)	1.98 (1.13, 3.44)*	28 (19)	1.82 (0.96, 3.46)
ASGC category (Remoteness):					
Inner regional	764	139 (18)		103 (13)	
Outer regional	787	129 (16)	0.85 (0.59, 1.21)	89 (11)	0.79 (0.52, 1.19)
Remote	396	67 (17)	0.90 (0.58, 1.40)	32 (8.1)	0.53 (0.30, 0.94)*
Very remote	154	28 (18)	0.90 (0.49, 1.66)	17 (11)	0.67 (0.32, 1.41)
Financial position:					
Prosperous/comfortable	336	40 (12)		26 (7.7)	
Reasonable	1069	152 (14)	1.20 (0.73, 1.97)	107 (10)	1.25 (0.68, 2.28)
Just getting along to very poor	696	171 (25)	2.23 (1.34, 3.70)**	108 (16)	1.99 (1.07, 3.69)*

*Note*: Based on a series of hierarchical logistic regressions, in which socio-demographic characteristics were entered simultaneously at step 1 (Table 3), followed by each of the current health status variables separately at step 2 (Table 4): * p < 0.01; ** p < 0.001. Only participants with the full set of socio-demographic characteristics were included. OR: Odds Ratio; CI: Confidence Interval.

The pattern of seeking help from non-professionals differed somewhat from that for professional contacts. Older age (65+) was associated with lower help seeking from non-professionals (OR = 0.34), as was residence in a remote area (OR = 0.53), while female gender was associated with proportionately higher help seeking from non-professionals (OR = 1.79). As with professionals, seeking help from non-professionals was also higher for those who were divorced or separated (OR = 2.29) and those whose financial position was “just getting along to very poor” (OR = 1.99).

Relationships between the selected measures of current health and reported professional and non-professional contacts for mental health problems during the past 12 months, after controlling for socio-demographic factor, shows proportionate increases in the odds of seeking professional (OR 1.95 to 11.5) and non-professional help (OR 1.70 to 10.4) across each successive category of poorer health or increased health risk (see Table 4). Severity of alcohol related problems on the AUDIT was the only health status characteristic not associated with professional contacts. For non-professional contacts smoking status was the only characteristic not associated with non-professional contacts. Three characteristics (poor/fair overall mental health, K-10 of 25 or higher, and more than five recent adverse life events) were each associated with at least five-fold increases in the relative odds of professional or non-professional contacts for mental health problems during the past 12 months.

**Table 4:** **Relationships between current health status characteristics and reported *professional *and *non-professional *contacts for mental health problems (N = 2101)**

**Health status characteristic**	**Sub-group n**	**Professional service utilisation**		**Non-professional service utilisation**	
		**n (%)**	**OR (99% CI)**	**n (%)**	**OR (99% CI)**
Overall mental health:					
Good to excellent [0]	1825	223 (12)		145 (7.9)	
Poor/fair [3]	273	140 (51)	6.49 (4.47, 9.43)**	95 (35)	5.55 (3.64, 8.45)**
Overall physical health:					
Good to excellent [0]	1671	242 (14)		161 (9.6)	
Poor/fair [1]	426	121 (28)	2.25 (1.59, 3.18)**	79 (19)	2.33 (1.54, 3.52)**
Kessler K-10 (current distress) score:					
10-15 [0]	1481	145 (9.8)		89 (6.0)	
16-24 [1]	478	139 (29)	3.43 (2.41, 4.88)**	98 (21)	3.61 (2.37, 5.49)**
25-50 [3]	131	77 (59)	11.5 (6.80, 19.5)**	54 (41)	10.4 (5.87, 18.5)**
AUDIT:					
0-7 [0]	1785	294 (16)		191 (11)	
8+ [1]	294	65 (22)	1.40 (0.91, 2.14)	47 (16)	1.70 (1.03, 2.78)*
PHQ-9 symptomatology:					
No symptoms [0]	1686	213 (13)		137 (8.1)	
Depressive symptoms [2]	400	147 (37)	3.62 (2.58, 5.07)**	104 (26)	3.77 (2.54, 5.58)**
Recent adverse life events:					
0-2 [0]	1644	223 (14)		131 (8.0)	
3-5 [1]	359	96 (27)	2.12 (1.47, 3.06)**	80 (22)	3.06 (2.02, 4.64)**
>5 [3]	46	29 (63)	7.74 (3.36, 17.8)**	19 (41)	5.63 (2.40, 13.2)**
Smoking status:					
No [0]	1820	283 (16)		191 (10)	
Yes [1]	265	78 (29)	1.95 (1.31, 2.91)**	47 (18)	1.53 (0.95, 2.48)

*Note*: Based on a series of hierarchical logistic regressions, in which socio-demographic characteristics were entered simultaneously at step 1 (Table 3), followed by each of the current health status variables separately at step 2 (Table 4): * p < 0.01; ** p < 0.001. Only participants with the full set of socio-demographic characteristics were included. OR: Odds Ratio; CI: Confidence Interval. Values in square brackets in the left-hand column reflect the weight given to each response category in calculating a Provisional Service Need Index.

### Potential needs, service adequacy and barriers

In the absence of detailed survey questions about met and unmet needs for specific professional services, we used the analyses reported in Table 4 to construct a simple Provisional Service Need Index (ranging from 0 to 14, where a higher score indicates a higher need). Health status characteristics associated with a less than 20% rate of professional help seeking received a weight of zero, while those with rates between 20% to 34%, 35% to 49%, and 50+%, received weights of 1, 2 and 3, respectively. For all participants who completed the service utilisation questions (n = 2150), the mean Provisional Service Need Index score was 1.89 (median = 1.00, SD = 2.64, distribution = 0: 40.2%; 1: 24.4%; 2–5: 24.0%; 6–9: 8.8%; 10+: 2.6%). A Receiver Operating Characteristic (ROC) analysis, with index scores as the predictor and professional help seeking status as the outcome, suggested that a cut-off point > 1 on the index optimised sensitivity (68%) and specificity (71%); with an area under the curve (AUC) of 0.74 (99% CI 0.70 to 0.78). Accordingly, we categorised scores on the Provisional Service Need Index as Low (0–1), Moderate (2–5), or High (>5), with assignment to the latter category requiring multiple health status characteristics with non-zero weights, at least one of which was weighted two or three.

Table 5 summarises the relationship between our provisional estimate of service need (based on aggregated current health characteristics) and reported contacts for mental health problems during the past 12 months; this approach is likely to be useful because it partially accounts for the overlap between the selected health characteristics. Participants assigned to the low need category had approximately half the rate of service contacts compared with the whole sample reported in Table 2 (professional: 8.5% vs. 17.3%; non-professional: 5.7% vs. 11.6%). By comparison, after controlling for socio-demographic characteristics, participants in the high need category displayed approximately ten-fold increases in the relative odds of seeking professional (OR = 10.5) or non-professional contacts (OR = 9.61).

**Table 5:** **Aggregate current health needs**^**¥ **^**and reported *professional *and *non-professional *contacts for mental health problems (N = 2101)**

**Provisional service need index**	**Sub-group n**	**Professional service utilisation**		**Non-professional service utilisation**	
		**n (%)**	**OR (99% CI)**	**n (%)**	**OR (99% CI)**
Low (0 – 1)	1359	116 (8.5)		78 (5.7)	
Medium (2 – 5)	504	121 (24)	3.29 (2.26, 4.78)**	72 (14)	2.78 (1.76, 4.40)**
High (>5)	238	126 (53)	10.5 (6.80, 16.3)**	91 (38)	9.61 (5.86, 15.7)**

*Note*: Based on hierarchical logistic regressions, controlling for socio-demographic characteristics (Table 3): * p < 0.01; ** p < 0.001. Scores on the Provisional Service Need Index are based on the seven health status characteristics identified earlier and their associated weights (Table 4). OR: Odds Ratio; CI: Confidence Interval. ^¥^ As reflected in the Provisional Service Need Index.

Among the 372 participants reporting any professional contacts for mental health problems, 300 (81%) provided data about the forms of help received and their overall adequacy. The majority of these (214 or 71.3%) indicated that they got as much help as they needed. A subset of participants (N = 89) also completed ratings about the factors that might have stopped or delayed their ability to get health care, the four main reasons being: the time taken to get an appointment (52%); distance to travel (45%); limited choices (42%); and cost (36%).

Table 6 presents a breakdown of the evaluations of service adequacy by the selected health status characteristics and the aggregate service need index. Perceived rates of ‘inadequate help’ ranged from 14% to 29% across the various reference categories, compared with up to 46% (OR 2.47-5.23). In short, poorer mental health and a higher number of recent adverse events were significantly associated with a higher likelihood of perceiving the help received as inadequate; physical health, severity of alcohol related problems, and smoking status were not associated with the perceived adequacy of help received.

**Table 6:** **Overall perceptions of help received among those reporting any *professional *contacts for mental health problems (N = 300)**

**Health status characteristic**	**Sub-group n**	**Inadequate help: did not get “as much help as you needed”**	
		**n (%)**	**OR (99% CI)**
Overall mental health:			
Good to excellent	168	34 (20)	
Poor/fair	132	52 (39)	2.47 (1.22, 4.99)**
Overall physical health:			
Good to excellent	191	46 (24)	
Poor/fair	109	40 (37)	1.83 (0.88, 3.84)
Kessler K-10 (current distress) score:			
10-15	104	15 (14)	
16-24	123	37 (30)	2.68 (1.07, 6.66)*
25-50	72	33 (46)	5.23 (1.95, 14.0)**
AUDIT:			
0-7	237	68 (29)	
8+	59	17 (29)	0.94 (0.38, 2.33)
PHQ-9 symptomatology:			
No symptoms	168	32 (19)	
Depressive symptoms	131	53 (40)	2.82 (1.37, 5.83)**
Recent adverse life events:			
0-2	172	36 (21)	
3-5	89	33 (37)	2.59 (1.16, 5.77)*
>5	26	12 (46)	3.59 (1.08, 12.0)*
Smoking status:			
No	227	59 (26)	
Yes	71	27 (38)	1.46 (0.66, 3.21)
Provisional Service Need Index^:			
Low (0 – 1)	80	11 (14)	
Medium (2 – 5)	101	23 (23)	1.77 (0.60, 5.18)
High (>5)	119	52 (44)	4.57 (1.68, 12.4)**

*Note*: Based on a parallel series of hierarchical logistic regressions to those reported in Table 4: * p < 0.01; ** p < 0.001. Scores on the Provisional Service Need Index are based on the seven health status characteristics and their associated weights (Table 4). OR: Odds Ratio; CI: Confidence Interval. Only includes participants who completed the survey section about the forms of help received and their overall adequacy.

## Discussion

### Overview of findings

Collectively, the rates and associations reported in Tables 2 to 6 provide a useful snapshot of service utilisation within our rural cohort, especially when viewed against the backdrop of the 2007 Australian NSMHWB findings (e.g., [5,10]). Our overall rate of professional contacts for mental health problems during the past 12 months exceeded the national rate (17% vs. 11.9%) [5], although gender effects were minimal in our data (males: 16% vs. females: 18%) compared with the national data (males: 8.8% vs. females: 14.9%). National contact rates with psychiatrists (2.3% vs. 2.5%) and psychologists (3.5% vs. 3.8%) were similar to our rates, however, there were lower contact rates for GPs in the national data (8.1% vs. 12%), which may reflect the younger profile of the national sample (64% vs. 41% aged under 55 years).

The findings reported in Tables 3 and 4 suggest that higher levels of help seeking tend to be associated with the absence of a partner, poorer financial circumstances, severity of current mental health problems, and higher levels of adversity, none of which are surprising associations. In the 2007 Australian NSMHWB, urbanicity was not significantly associated with service usage [6] and in the current study remoteness was only modestly associated with a lower likelihood of accessing non-professional supports.

In the 2007 Australian NSMHWB (see Table 4 of reference [10]), almost all participants (96.5%) who reported using ambulatory care services for mental health problems in the past 12 months also reported an overall need for care (on the Perceived Need for Care Questionnaire); however, 17.8% of those with an expressed need for care did not report any service use. Conversely, only a small percentage of non-users of those services (2.8%) reported any need for care. Therefore, in the current study, mental health service usage status is probably a reasonable proxy for perceived need status, and, if anything, is likely to be an underestimate. Here, 8.5% of participants with an estimated low service need nevertheless reported using professional services (see Table 5), which compares favourably with the 6.5% of national survey participants without a 12 month mental disorder who reported a need for care (see [10], page 630). In the current study, 53% of those with an estimated high service need actually reported using professional services (see Table 5), which falls between the national survey’s perceived need for care rates for participants with any 12 month mental disorder (43.3%) and those with any 12 month affective disorder (70.1%) (see [10], page 630). Our study and the national survey [5] revealed a dose–response relationship between the severity of mental health problems and the likelihood of seeking any professional or non-professional help. Finally, in the national survey, among users of ambulatory mental health services (who also expressed a need for care), 54.1% indicated that their needs were fully met and 44.8% that they were partially met (see [10], page 631), which is similar to the 44% of those with high need in this analysis who indicated that they did not get as much help as needed ([10], page 632).

### Implications and research directions

The rates of professional contacts are encouraging in the light of the specialist workforce shortages in rural and remote regions, nevertheless perceptions of adequacy of care should concern clinicians and policy makers. Similar numbers of professional contacts by males and females suggests that a number of “men’s health” campaigns that have been conducted Australia-wide may be increasing recognition and treatment acceptability among men with mental health problems. Various programs of fly-in psychiatry services [52] and subsidised access to psychologists [23] designed to improve access to specialist mental health care in rural regions may have also contributed to this finding. The low levels of contacts with Drug and Alcohol Counsellors, mental health nurses and other mental health professionals (e.g. social workers) is of concern and is likely to be related to supply shortages in these communities [53]. We have addressed the issue of alcohol usage and problems in detail elsewhere [54].

Most rural and remote residents have access to primary health care and GPs whether resident or outreach. The GP has a central role as provider of care for this population and as a means of referral. Attendance at a GP surgery does not carry the stigma of consulting a psychiatrist or psychologist, particularly in small communities [24]. While GP and specialist services are funded separately in the Australian health care system, this does not diminish the need for collaboration and the role of the newly established regional primary health care organisations (Medicare Locals) is key to achieving more integrated care with other providers of mental health services [55].

Non-professional help seeking seems lower than that reported by Oleson [6] and was significantly lower for those aged over 65. Service use cannot be understood as a proxy for health outcomes and timely contacts with skilled practitioners may have better outcomes than multiple contacts with others who may not have the time or the experience to provide appropriate help.

The importance of the three predictors of help seeking which were strongly associated with high service need (namely: absence of partner; poor financial circumstances; and number of adverse effects) is not surprising; nonetheless, awareness of these potential influences is important for primary mental health providers and for increasing mental health literacy in rural and remote communities, a responsibility shared between Australian governments, and programs such as the National Depression Initiative [56].

The association between service need and service use is similar to the findings of the 2007 Australian NSMHWB [10] and suggests that targeting of services to high needs clients is similar for rural residents. Nevertheless, we need to know more about those who appear to have high needs but do not use services. A lack of service use in the face of high need may relate to health service factors (insufficient quantity, effectiveness or appropriateness) [5], community level factors (stigma) [57], individual factors (stoicism) [24] or even environmental factors such as unemployment, chronic financial strain and environmental adversity such as drought (a feature of many rural areas through the period of this study) [58,59]. The barriers to service use such as delays in appointments, distance, limitations of choice and greater cost are not surprising but may be amenable to telephone or video consultations and interventions.

### Study limitations

This analysis is based on cross-sectional data and we cannot be certain about the sequence of events. As with all self-reported data, there is the possibility of over- or under-reporting of service use, and the overall participation rate indicates caveats regarding generalizability of the data. While the response rate to ARMHS at baseline is low (27%), it matches other population based surveys in rural communities [24,60] and those using telephone based recruitment [61]. It is possible that participants with contact with specialist mental health services (and, therefore, potentially more inclined to respond to such a survey) were over represented, hence partially explaining the findings regarding higher professional service use in our sample. The study does not examine types or effectiveness of interventions offered based on clinical severity. Longitudinal data from this study at subsequent waves of data collection will provide the opportunity to investigate such questions.

The current paper contributes to the reduction of an important gap in the evidence base, namely the under-representation of rural and particularly remote residents in national mental health surveys. As the first stage of a longitudinal cohort study, further analyses at three years and five years will examine patterns of service use and mental health outcomes at individual (including mental health diagnosis and severity), household and aggregate levels and in relation to the range of predictors discussed above.

## Conclusions

This study examined self-reported patterns of professional and non-professional service use for mental health problems in a rural community cohort. The findings indicate relatively high rates of GP attendance for such problems compared with data from metropolitan settings. This suggests that appropriate support for rural and remote GPs for their role in mental health care should be a priority. Marital status and financial factors, along with recent adversity and current distress levels were associated with service use. Although participation rates limit the conclusions that can be drawn regarding overall population use of services, relationships between a measure of Provisional Service Need and service use indicated that those with higher needs were more likely to access specialist services. This is encouraging for service providers. Nevertheless, geographic and financial barriers to service use were identified and perception of service adequacy was relatively low among those with highest levels of distress and greatest adversity.

## Abbreviations

ARMHS: Australian rural mental health study; ARIA+: Accessibility/remoteness index of Australia plus; ASGC: Australian standard geographic classification; AUC: Area under the curve; AUDIT: Alcohol use disorders identification test; CI: Confidence interval; CIDI: Composite international diagnostic interview; GP: General practitioner; K-10: Kessler 10 measure of psychological distress; NSMHWB: National Survey of Mental Health and Wellbeing; NSW: New South Wales; OR: Odds ratio; PHQ-9: Patient health questionnaire – nine item; ROC: Receiver operating characteristic; TICS-M: Telephone interview for cognitive status; UK: United Kingdom; US: United States

## Competing interests

The authors declare that they have no competing interests.

## Authors’ contributions

DP led the writing of the paper, TL devised the Provisional Service Need Index and led the data analysis with MF, CC managed the data collection and custodianship, BK had the original idea for the study and is chief investigator, while the remaining authors JF, KI, JA, DA, RR and RB took a full part in the drafting and development of the paper. All authors read and approved the final manucripts.

## Pre-publication history

The pre-publication history for this paper can be accessed here:

http://www.biomedcentral.com/1472-6963/13/157/prepub
